# Ketamine in post-stroke depression: a report of 2 cases

**DOI:** 10.3389/fpsyt.2025.1680687

**Published:** 2025-12-04

**Authors:** Adam Włodarczyk, Jakub Słupski, Joanna Szarmach, Dominika Milewska, Wiesław J. Cubała

**Affiliations:** Department of Psychiatry, Faculty of Medicine, Medical University of Gdańsk, Gdańsk, Poland

**Keywords:** post-stroke depression, ketamine, safety, tolerability, case report

## Abstract

**Background:**

Post-stroke depression (PSD) is a common comorbidity following a stroke, often leading to significant emotional and physical disability. While antidepressant medications can alleviate depressive symptoms, treatment resistance is frequently observed in PSD patients. Ketamine, an N-methyl-D-aspartate (NMDA) receptor antagonist, has shown potential as a treatment for depression due to its neuroprotective, anti-inflammatory, and psychoplastogenic effects. Its effectiveness in treatment-resistant post-stroke depression (TRD-PSD) remains underexplored.

**Methods:**

Two patients diagnosed with treatment-resistant post-stroke depression were treated with intravenous ketamine. Ketamine infusions were administered over a 4-week period in both cases, and the patients’ depressive symptoms were closely monitored.

**Results:**

Both patients demonstrated improvement in depressive symptoms following ketamine treatment. The treatment was well tolerated, with minimal side effects reported, and no significant adverse events were observed during the treatment course.

**Conclusions:**

Ketamine may be a viable treatment option for patients with treatment-resistant post-stroke depression. Further research is warranted to better understand the efficacy and safety of ketamine in this specific patient population.

## Highlights

- Minimal adverse events observed in two patients with post-stroke depression.
- Intravenous ketamine was safely administered over 4 weeks.
- Ketamine may offer neuroprotective effects for post-stroke depression.
- Further studies are needed to assess ketamine’s long-term safety in PSD.

## 1 Introduction

Globally, stroke ranks as the second leading cause of mortality and occupies the third position in terms of its impact on disability-adjusted life years (DALYs). This not only highlights the profound health burden that stroke imposes worldwide but also underscores the critical need for enhanced preventive measures, early detection, and innovative treatment strategies to mitigate its devastating effects (1).

Post-stroke depression (PSD) is a common comorbidity post-stroke, reaching from 18 to 42% of post-stroke patients (2–4). Due to the symptomatic overlap between stroke and depression, PSD is often inaccurately diagnosed at an early stage of post-stroke comprehensive treatment, affecting patients’ functional and social outcomes. Consequently, it is essential to employ the diverse diagnostic approaches aforementioned for the early detection of PSD. Some antidepressant medications have been shown to ameliorate symptoms of depression (5–7). However, refractoriness is commonly observed.

Ketamine, an N-methyl-D-aspartate (NMDA) receptor antagonist, is the prototypal glutamatergic agent with demonstrated efficacy in treatment-resistant depression (TRD) and suicidality (8, 9). Its most common side effects are psychotomimetic and dissociative symptoms (10). Previous research has shown that ketamine has the property of being rapid-acting with a reduction in suicidality (11). The findings underscored promise for the focus on the development and use for rapid-acting antidepressants.

There is lacking data on rapid-acting antidepressants (12) in PSD. Scarce data available shows that ketamine could be a safe option (10, 13, 14). However, ketamine, through NMDA receptor (NMDAR) antagonism, can increase cerebral blood flow (CBF), cerebral metabolism (CMRO_2_), and intracranial pressure (ICP), particularly under conditions of spontaneous ventilation or pre-existing cerebrovascular dilation. Although some data suggest potential neuroprotective effects, these hemodynamic changes may exacerbate intracranial hypertension or compromise autoregulation, posing significant risks in vulnerable patients. For this reason, individuals with recent cerebrovascular events are typically excluded from clinical trials, as ketamine’s cerebrovascular effects could aggravate neurological injury (15).

## 2 Methods and population

In this paper, we use TRD definition according to the definition adopted by the European Medicines Agency (EMA) and the U.S. Food and Drug Administration (FDA), where TRD is defined as the lack of efficacy of at least two different antidepressant medications administered at adequate doses and for an appropriate duration during the current major depressive episode (MDE) in patients with major depressive disorder (MDD) (16, 17). Unlike the FDA definition, the EMA explicitly states that the ineffective antidepressants may belong either to the same or to different pharmacological classes (16). The diagnosis of post-stroke depression was based on a structured clinical evaluation conducted by a board-certified psychiatrist, using ICD-10 diagnostic criteria. The temporal association with a confirmed cerebrovascular event, persistence of mood and neurovegetative symptoms beyond normal post-stroke adjustment, and exclusion of alternative neurological or medical explanations were all considered. Symptom severity was assessed using validated scales, and is discussed in detail elsewhere (10, 13, 14).

Two patients reported as PSD specifier in TRD described in the manuscript originate from the safety and tolerability registry for the tertiary reference unit for mood disorders and apply to short-term intravenous ketamine use as the add-on to the standard of care treatment administered across 8 ketamine infusion over 4 weeks, dosed at 0.5 mg/kg over 40 minutes. The registry is described in detail elsewhere (10, 13, 14).

All subjects gave written informed consent to participate in the study. The study was carried out in accordance with the latest version of the Declaration of Helsinki (NKBBN/172-674/2019) and registered as NCT04226963, specifying ketamine as the investigational treatment.

## 3 Study design: ketamine infusions

The study design was discussed in detail elsewhere (10, 13, 14). In this observational study, in which patients continued their standard-of-care medication at baseline and underwent 8 ketamine infusions over 4 weeks. Ketamine was dosed at 0.5 mg/kg and diluted into 0.9% saline solution administered over 40 minutes. Safety monitoring was done by the study clinician before, during, and post-infusion up to 90 minutes post-infusion at 15-minute intervals. Safety, including vital signs, mental status, and adverse events [as defined by European Medicines Agency (18, 19)], was monitored and recorded every 15 min. The ECG was carried out before every second infusion and one week after the last ketamine infusion. Investigators were advised to monitor BP on treatment days as a safety measure (i.e., no dosing if predose systolic RR >140 mmHg (>150 for age >65 years) or diastolic RR >90 mmHg, and dose interruption if postdose systolic RR ≥200 mmHg (≥190 for age >65 years) or diastolic RR ≥110 mmHg (≥100 for age > 65 years) subjects with high RRs were referred to the respective specialist for evaluation and eventual treatment. Blood pressure and heart rate were measured using a certified fully automated device before dosing (0 minutes) and at 15, 40, 45, 60, and 90 minutes postdosing, after at least 5 minutes of rest in the supine position.

## 4 Case series

### Case 1

The initial case study presents a 49-year-old Caucasian male. The patient came from an intact family; the mother suffered from depression. During the interview, the patient denied experiencing any traumatic events. At the time of hospital admission was receiving a disability pension due to a past stroke. Previously, worked in an administrative position. The patient’s financial situation was good at the time of hospitalization, and he was living with his wife and one child. The patient has a medical history significant for arterial hypertension, hypercholesterolemia, hypothyroidism, and ischemic heart disease. At 40 years of age, he experienced an ischemic stroke involving the left middle cerebral artery territory, contributing to the left frontal cortical region, had lenticulocapsular infarcts and lesions localized to the left basal ganglia area. Details of the patient’s current medication regimen are provided in **Table 1**.

**Table 1:** Concomitant pharmacotherapy in reported cases.

Case	Time point	Psychiatric medications (daily dose)	Somatic medications (daily dose)
Case 1 (49-year-old male)	Before ketamine (first series)	1^st^ line of treatment: Moclobemide 450 mg, 2^nd^ line of treatment: Sertraline 200 mg. 3^rd^ line of treatment: Clomipramine SR 150 mg (later ↑ to 300 mg), Quetiapine IR 125 mg,	Levothyroxine 50 µg, Cilazapril 5 mg, Bisoprolol 5 mg, Verapamil hydrochloride 80 mg, Fenofibrate 215 mg, Atorvastatin 20 mg, Acetylsalicylic acid 75 mg, Piracetam 1.2 g, pregabalin 150 mg, clonazepam 1 mg
	Additional trials (before ketamine)	Reboxetine up to 12 mg, Lithium up to 750 mg	—
	At readmission (second series)	Escitalopram 20 mg, Quetiapine IR 200 mg, Pregabalin 300 mg, Clonazepam 1 mg	(same as above)
Case 2 (60-year-old male)	Before ketamine	1^st^ line of treatment: Fluoxetine 40 mg, Trazodone 150 mg, 2^nd^ line of treatment: Paroxetine 40 mg, 3^rd^ line of treatment: Duloxetine 60 mg, Quetiapine XR 200 mg, 4^th^ line of treatment: Clomipramine SR 225 mg, Quetiapine XR 300 mg, Quetiapine IR 100 mg, Clorazepate 5 mg, Folic acid 15 mg	Hydrochlorothiazide + Losartan 100 + 25 mg, Acetylsalicyc acid 150 mg, Amlodipine 5 mg, Methotrexate 5 mg once weekly

Following a stroke the patient suffered from cognitive impairment, left lower limb paresis, right hand tremor, diminished facial expressiveness, impaired memory and attention, decreased sensation of the right side of the face, and weakness in elevation of the right shoulder and shoulder joint. Subsequently, he developed a depressive syndrome and received a diagnosis of a major depressive episode. From the onset of his depression to his enrollment in the study, he required hospitalization six times for recurrent depression. During the specified episode, the patient presented with a severe depressive syndrome characterized by markedly lowered mood and psychomotor activity, pronounced lassitude, anhedonia (Snaith–Hamilton Pleasure Scale score (SHAPS) = 11), sleep disturbances, diminished appetite, and cognitive impairment that interfered with conversation and social interaction. He displayed a resigned attitude and reported suicidal thoughts accompanied by a formulated suicidal plan. His treatment regimen at that time included sustained-release clomipramine 150 mg/day, pregabalin 150 mg/day, immediate-release quetiapine 125 mg/day, and clonazepam 1 mg/day. Prior to ketamine administration, the patient underwent three electroconvulsive therapy sessions but was precluded from further treatments due to elevated blood pressure and respiratory challenges. The clomipramine dosage was subsequently increased to 300 mg/day. The medication was then changed to reboxetine at a dose of up to 12 mg/day, supplemented with lithium at a maximum of 750 mg/day, yet no clinical improvement was noted. The patient was then administered eight intravenous infusions of ketamine at a dosage of 0.5 mg/kg. Throughout the treatment period, both the central nervous system and cardiovascular safety were monitored following the study protocol. No severe adverse events were reported, with the most common central nervous system adverse effects being transient and fully resolving dissociative symptoms. In terms of cardiovascular safety, the elevated respiratory rate and blood pressure levels normalized within approximately 1.5 hours. The Montgomery–Åsberg Depression Rating Scale (MADRS) score was 38 on admission and 35 at discharge (with no suicidal risk) to a psychiatric daily ward. Self-report by the patient measured in The Inventory of Depressive Symptomatology (IDS-30-SR) scale respectively: 62 to 68.

Six months after discharge, the patient was readmitted due to a relapse of depressive symptoms, similar to those from the first paragraph. The sole adverse effect reported between the two hospitalizations was hand tremors, likely attributable to lithium carbonate, which was gradually discontinued as a result. Upon admission, the patient’s treatment consisted of escitalopram at 20 mg/day, immediate-release quetiapine at 200 mg/day, pregabalin at 300 mg/day, and clonazepam at 1 mg/day. The patient was deemed eligible for a second series of ketamine infusions and, as previously, received eight intravenous doses of ketamine at 0.5 mg/kg. Consistent with the earlier treatment, no central nervous system or cardiovascular adverse events were noted. Observed increases in arterial pressure and heart rate, along with dissociative symptoms, subsided swiftly without causing harm. This time, the ketamine effect was significant: the MADRS score decreased from 40 to 18, with IDS-30-SR 65 to 50. No sequelae of ketamine administration in safety and tolerability readout appeared in 12-month-long follow-up.

### Case 2

The second case study pertains to a 60-year-old Caucasian male. The patient came from an intact family, with no family history of mental illness, and denied any significant traumatic experiences in his personal life. On admission, he was receiving a disability pension due to a previous stroke and depression; previously worked in the construction industry. The patient described his financial situation as satisfactory and was living in a flat with his wife at the time of admission. He had two children who no longer lived with them. His medical history was significant for arterial hypertension and rheumatoid arthritis. Details of current medications are provided in **Table 1**. Diagnosed and treated for MDD and obsessive-compulsive disorder since the age of 47. He experienced two transient ischemic attacks at ages 52 and 56 involving the left middle cerebral artery territory, contributing to the left frontal cortical region, and the left orbital part of the inferior frontal gyrus. The patient developed post-stroke symptoms, including reduced facial expressiveness, hand tremor, short-stepped, shuffling gait. The episode in question marked the patient’s fifth depressive episode. This episode was characterized by treatment resistance, which justified the diagnosis of TRD, distinguishing it from the previous depressive episodes that had occurred prior to the strokes. Considering the emergence of treatment resistance as a novel phenomenon, and in the absence of other identifiable factors that could have influenced the course of the illness and its symptomatology, a diagnosis of PSD was established. At admission, the patient presented with a depressed mood and diminished motivation. He reported reduced energy, hypersomnia, and increased appetite, accompanied by significant cognitive impairment and impaired attention and concentration. Anhedonia (SHAPS = 9). His self-esteem was lowered, and he experienced fleeting suicidal thoughts without a specific plan. Before the initiation of ketamine infusions, patient had undergone 18 sessions of electroconvulsive therapy, which failed to yield a satisfactory response. Upon admission, the patient’s pharmacotherapy included sustained-release clomipramine at 225 mg/day, extended-release quetiapine at 300 mg/day, immediate-release quetiapine at 100 mg/day, clorazepate at 5 mg/day, and folic acid at 15 mg/day. He was enrolled in the study and administered 19 intravenous doses of ketamine at a dosage of 0.5 mg/kg. The effectiveness of ketamine treatment, assessed using MADRS, showed a reduction in depressive symptom severity from a score of 27 at admission to 21 at discharge. Self-report by the patient measured in IDS-30-SR scale were 59 upon admission to 55 at discharge. Monitoring of cardiovascular and central nervous system safety was conducted in alignment with the study protocol. In concordance with the previous case, no serious adverse events were reported. No serious adverse events were reported in 12-month-long follow up.

None of the patients achieved remission, but both showed a reduction in Clinical Global Impression–Severity (CGI-S) scores. The first patients’ depression improved from 6 (“Severely ill”) to 3 (“Moderately ill”), and the second patient from 5 (“Markedly ill”) to 4 (“Moderately ill”).

Self-report scales may not accurately reflect the severity of illness in mentioned patients because their scores are substantially influenced by post-stroke neurological symptomatology and reduced quality of life.

In both cases transient elevations in blood pressure were observed on ketamine treatment days, with peak values occurring approximately 40 minutes after the start of administration and returning to predose levels thereafter (not exceeding 14- and 17-mm Hg, which represented the greatest deviations observed during ketamine infusion in the first patient during the first and third administrations successively). None of the patients experienced treatment-emergent transient hypertension (defined as a systolic blood pressure ≥180 mm Hg and/or a diastolic blood pressure ≥110 mm Hg). No clinically significant electrocardiographic abnormalities (including tachycardia) have been reported.

None of the patients had neurocognitive deterioration in the course of the treatment.

## 5 Discussion

The 2 cases described demonstrate a favorable safety and tolerability profile in short-term ketamine use in PSD. The existing data from research shows that the prevention of PSD is complicated by its pleiotropic symptomatology, underscoring the need for more structured randomized controlled trials, to determine the safety and effectiveness of both pharmacological and non-pharmacological, as well as integrative prevention strategies aimed at reducing PSD incidence among stroke patients.

PSD is associated with infarcts involving the left frontal cortical region, left basal ganglia, and brainstem. These findings are partially consistent with earlier studies highlighting the importance of lesion proximity to the left frontal pole, and implicating prefrontal and basal ganglia structures in the development of PSD. A left-sided stroke lesion has been identified as a factor contributing to early-onset PSD, while thalamic lesions have been significantly associated with PSD in the acute stage of stroke. Moreover, left lenticulocapsular infarcts were shown to be an independent predictor of depressive symptoms 1 month after stroke onset (20). Notably, several of these observations align with the lesion locations in our case reports, in which strokes predominantly involved left frontal and basal ganglia regions, as described above.

PSD is associated with reduced central concentrations of the biogenic monoamines serotonin, dopaminę, and norepinephrine. The principal monoaminergic nuclei are located in the brainstem, with ascending projections to widespread brain regions, including the cortex and limbic system. Ischemic stroke can disrupt these projections from the midbrain and brainstem, thereby diminishing the bioavailability of serotonin, noradrenaline and dopamine. Such lesions may damage axons transmitting biogenic amines from the brainstem to the cerebral cortex, leading to decreased monoamine levels in the frontal and temporal lobes, as well as in peripheral tissues and the basal ganglia. Consistent with the classical monoamine hypothesis, these changes support the notion that depression is linked to reduced levels of monoamines, particularly 5-HT, NE, and DA (21, 22).

In preclinical models of PSD, middle cerebral artery occlusion (MCAO), modulation of small-conductance calcium-activated potassium (SK) channels within the ventral tegmental area (VTA) has been shown to influence depressive-like behaviors. Stimulation of SK channels enhances the expression of dopaminergic neurons in the VTA of PSD rats and is associated with a worsening of depression-related behaviors, whereas pharmacological inhibition of SK channels reduces dopaminergic neuronal expression and alleviates these behaviors. Moreover, MCAO-induced reductions in the density of VTA dopaminergic neurons are closely linked to decreased brain dopamine concentrations, which in turn correlate with the emergence of depression-like symptoms during the middle stage of stroke in neonatal rat models. Unilateral ischemic lesions in the medial prefrontal cortex lessen the innervation of serotonin and noradrenaline in the affected area, as well as in other brain regions distal to the stroke (21).

Tailored non-pharmacological interventions (including educational, mental, and physical health support) in combination with pharmacological measures represent the most viable strategy for PSD prevention and treatment (6), although ketamine may seem an option.

Regarding PSD, ketamine might be considered as a multifaceted agent offering neuroprotective, anti-inflammatory, and psychoplastogenic effects. Ketamine exerts its effects through mechanisms distinct from conventional antidepressants, most of which rely on modulation of monoaminergic transmission and, in clinical practice, are predominantly selective serotonin reuptake inhibitors. Its neuroprotective properties may stem from its ability to mitigate glutamate excitotoxicity and foster synaptic repair, crucial in the aftermath of cerebrovascular events (23). Although ketamine is most commonly characterized as a NMDAR antagonist that modulates glutamatergic transmission, it also exerts pharmacological effects on opioid, monoaminergic, and cholinergic systems. Furthermore, emerging evidence indicates that certain ketamine metabolites may exert antidepressant effects independently of NMDAR antagonism (20). Thus, the mentioned disruptions in PSD could be addressed with ketamine. Anti-inflammatory actions are particularly pertinent given the established link between chronic inflammation and treatment refractoriness in PSD, as preclinical data suggest that sustained inflammatory responses may impede the efficacy of conventional antidepressants (24). Furthermore, ketamine’s psychoplastogenic potential is evidenced by its capacity to induce rapid synaptogenesis and facilitate neural plasticity, which may counteract the synaptic deficits often observed in PSD (25). Also, ketamine’s primary mechanism involves non-competitive antagonism of the NMDAR. By attenuating this receptor’s activity and shifting glutamatergic signaling toward greater AMPA transmission, ketamine promotes the release of brain-derived neurotrophic factor (BDNF). It facilitates synaptogenesis—the restoration of synaptic connectivity disrupted in major depressive disorder. Reduced BDNF expression has been consistently linked to the pathophysiology of MDD, and ketamine’s capacity to rapidly reverse these neurobiological deficits is central to its therapeutic profile. This reduces excessive glutamatergic signaling and disinhibits GABAergic interneurons, subsequently increasing AMPA-mediated transmission. By increasing AMPA throughput and engaging BDNF–TrkB signaling, ketamine triggers a surge in synaptogenesis and dendritic spine formation, particularly in the prefrontal cortex and hippocampus. Across conditions, this shift restores synaptic balance in circuits disrupted by chronic stress, trauma, or persistent nociceptive signaling (23).

Experimental and clinical data suggest that chronic post-ischemic states are characterized by sustained, dysregulated glutamatergic signaling, with relative overactivation of extrasynaptic NMDA receptors, calcium-dependent neurotoxic cascades, and impaired synaptic plasticity in fronto–subcortical and limbic networks implicated in mood regulation. In this scenario, NMDA receptor activity may be dominated by tonic, maladaptive “noise” rather than phasic, synapse-specific signaling necessary for adaptive circuit reorganization. Partial NMDA receptor antagonism with ketamine could, therefore, attenuate this pathological glutamatergic drive, shift transmission toward AMPA-mediated pathways, enhance BDNF-dependent synaptogenesis, and facilitate functional reorganization of post-stroke networks. From this perspective, the antidepressant effect of NMDA receptor antagonists in post-stroke, treatment-resistant depression and in depression associated with chronic pain may reflect a shared capacity to normalize aberrant downstream glutamatergic signaling (20, 22, 26).

Both academic and industry sponsored interventional studies on ketamine and its enantiomers use in subjects with mood disorders exclude subjects with either active psychiatric comorbidity or the history of cerebrovascular events (27). Above all it is true for regulatory trials, where the comorbidities are regarded as the confounding factor for the data interpretation. However, in the real-life setting subjects with the comorbidities represent a substantial proportion of patients with mood disorder in particular with TRD. As TRD is a relatively novel concept brought to the academic and industry studies, there is still the unmet need for the personalized definition of the affective episodes that fits real-world clinical setting. The variability of the definitions for the MDD produces a thought-provoking concept of treatment refractory depression, difficult to treat depression, chronic major depression. All of which do not substitute for the concept of TRD (28). Besides in line with ketamine’s SmPC the history of cerebrovascular events is a contradictory condition for ketamine use.

The use of ketamine cannot be recommended as the advantageous approach in PSD over standard-of-care (SoC) treatment. However, given that treatment chronicity and refractoriness are known predisposing factors for no response, the consideration of ketamine may be warranted in some patients when all other intervention measures are exhausted, provided these patients have stable somatic comorbidities and are under close safety oversight. Moreover, evidence indicates that ketamine may precipitate mood elevation in susceptible individuals (e.g. with bipolar disorder); however, the available clinical data suggest that this risk is low. In a recent scoping review of ketamine and esketamine for bipolar depression, including 10 clinical studies, Jawad et al. (29) reported that ketamine treatment was generally well tolerated and associated with only a minimal risk of manic/hypomanic switching. In our study, all participants met criteria for unipolar post-stroke depression, and patients with a history of bipolar disorder or prior manic/hypomanic episodes were excluded. During the ketamine treatment course and follow-up, patients were clinically monitored for emergent manic or hypomanic symptoms, and no manic/hypomanic switch was observed in our sample.

The main limitation of this report is its’ non-generalizability as it refers to the cases described. Moreover, there is no evidence for causality assessment. The two cases reported shall be interpreted as straightforward observations. This study concentrates explicitly on TRD in the course of both MDD and bipolar depression, with a proportion of the patients having physical comorbidities and concomitant medications. The safety and tolerability results require further investigation with larger samples and a longer study duration. While, of course, specific safety findings are applicable to TRD subjects showing a clinical response, at the current time, not all findings are displayed due to sample size considerations. Future more extensive studies, therefore, need to be mindful of this. Furthermore, our study had a short follow-up duration, around 12 months. The strengths of the current study are in the representation of the ecological clinical sample: they stand for the comorbidities and disease stages not represented within the regulatory trial, with a chronic/refractory course that may fall into a different entity. Thus, it provides a future study design on whether ketamine is useful and safe enough to be an accepted form of treatment as an acute treatment option in TRD. Further investigation is necessary to perform larger, more rigorous RCT designs.

## 6 Conclusion

The two cases described contribute to the literature on the ketamine favorable safety and tolerability in mood disorders. To our best knowledge it is the first report on ketamine use in PSD, that may contribute to the future study design and data interpretation. Above all, as the course of PSD is characterized by the delayed onset and poor long-term prognosis with currently available SoC, any safe and conclusive observation that may benefit patients’ well-being is of prime importance.

## Data Availability

The raw data supporting the conclusions of this article will be made available by the authors, without undue reservation.
